# Randomized Comparison of Hypochlorous Acid With 5% Sulfamylon Solution as Topical Therapy Following Skin Grafting

**Published:** 2019-05-31

**Authors:** Kevin N. Foster, K. J. Richey, J. S. Champagne, M. R. Matthews

**Affiliations:** The Arizona Burn Center at Maricopa Integrated Health System Phoenix

**Keywords:** hypochlorous acid, Sulfamylon, skin graft, healing, pain

## Abstract

**Objective:** Infections are a serious complication of thermal injury. Excision and grafting have led to a decrease in incidence, but to ensure successful skin grafting, antimicrobial irrigants are frequently utilized to prevent infection. A safe, efficacious, and cost-effective irrigant capable of preventing infections would be a valuable adjunctive therapy. The objectives of this study were to determine whether the test article was noninferior to current therapy in controlling infection and reducing postoperative pain in patients with skin graft. **Methods:** Patients with burns requiring skin grafting were randomized to hypochlorous acid or 5% Sulfamylon solution as topical dressings postoperatively. Inclusion criteria included thermal injury 20% or more total body surface area requiring excision and autografting, and age 18 years or more. Exclusion criteria included pregnant females, chlorine sensitivity, and electrical/chemical/cold injuries. The following outcomes were assessed: patient demographics, graft viability, infection, pain score, narcotic usage, adverse events, and cost. **Results:** Treatment groups were demographically equivalent. There were no differences in adverse or serious adverse events between the 2 groups. Graft viability and infection rate were equivalent between the 2 groups. In addition, pain scores and narcotic usage were similar. Hypochlorous acid was significantly less expensive than 5% Sulfamylon solution. **Conclusions:** Hypochlorous acid demonstrated equivalent efficacy and safety compared with 5% Sulfamylon when used as the postoperative topical dressing for skin grafts. Hypochlorous acid was more cost-effective. This pilot study was limited by its small sample size. However, hypochlorous acid shows promise as a topical wound dressing and further study with larger groups is warranted.

It is estimated that each year in the United States, approximately 500,000 individuals suffer from burns that that require medical attention. Annually, 4000 people die from burn injuries. In total, 40,000 patients with burns are hospitalized each year and 60% (25,000) of these patients are admitted to hospitals with specialized burn units.^1^

Patients with burns, due to the loss of protective skin layer and the immunosuppressive nature of thermal injury, are prone to infection. This is additionally true because most burn centers practice early burn excision and grafting.^2^^-^4 Thus, antimicrobial protection during every step of burn treatment, including surgical interventions, is considered necessary. Most burn centers use topical agents routinely over grafts because of this risk of infection, even in patients with smaller burns.

The causative agents of burn wound infections include bacteria such as *Pseudomonas aeruginosa*, *Klebsiella* sp, *Staphylococcus* sp, *Escherichia coli*, *Proteus mirabilis*, *Enterococcus* spp, *Enterobacter* spp, *Streptococcus* sp, and *Acinetobacter* sp and fungi such as *Aspergillus* sp, *Fusarium* sp, *Phycomycetes*, and *Candida* sp.^5^^-^8 But because of their immunocompromised status, patients with burns may be susceptible to infections with more unusual microorganisms, even those that normally are not virulent in healthy people. These bacteria might be platonic but often occur as a biofilm.^9^ Thus, antimicrobial agents to be used need to have a broad antimicrobial spectrum.

Commonly used topical antimicrobial agents include silver sulfadiazine cream, mafenide lotion, povidone-iodine solution and mafenide acetate 5% solution, Dakin's solution, and other antimicrobial creams, lotions, ointments, and solutions. Bandages, used to cover the grafts, typically must be kept moist for continuous protection and to avoid drying out of the grafts and the wound bed.

Many of these topical agents have adverse effects on healing. Both povidone-iodine and mafenide solution are known to be toxic to mammalian cells and thus might have a detrimental influence on wound healing.^10^^,^^11^ In addition, absorption of iodine may lead to systemic toxicity,^12^^,^^13^ whereas mafenide may be painful and lead to metabolic acidosis through inhibition of carbonic anhydrase.^14^ Allergic reactions to both materials have been described as well.

Thus, while topical antimicrobial therapy is necessary to protect against infection, agents used do have side effects; however, these are accepted because of the overwhelming need to protect a fresh graft and its wound bed.

Hypochlorous acid (HOCl) is a topical antimicrobial with many desirable characteristics. HOCl is produced in vivo by neutrophils as part of the respiratory burst pathway.^15^ This pathway plays a crucial role in intracellular killing of microorganisms by leukocytes.^16^^-^18

In in vitro studies, HOCl has been shown to rapidly kill gram-positive and gram-negative microorganisms, including methicillin-resistant *Staphylococcus aureus* and vancomycin-resistant *Enterococcus*.^19^ In additional preclinical studies, the compound was shown to produce greater than log_5_ kill within 5 minutes of contact against a wide range of pathogens.^20^ So far, microbial resistance to HOCl acid has not been identified.

HOCl is thought to have antimicrobial properties via a number of different mechanisms, including the inhibition of bacterial plasma membrane proteins involved in energy transduction^21^: this leads to loss of homeostatic control of ions across the membrane and causes cell swelling. Other hypotheses describe the oxidation of sulfhydryl protein moieties in the bacterial membrane.^22^^,^^23^

HOCl has no cellular toxicity to human cells when used in clinically effective dosage: mammalian bodies regulate the levels of HOCl during the inflammatory response using intrinsic antioxidant defense systems by using compounds such taurine and nitrites to neutralize HOCl and to protect against oxidative damage to cells.^24^^-^28

HOCl has been used extensively for surface disinfection, for the cleaning of endoscopes,^20^^,^^29^ and as a sanitation method to eliminate pathogenic organisms on foods and surfaces in food service areas.^30^

HOCl irrigation reduces bacterial counts in chronic open wounds more effectively than saline. HOCl irrigation in chronic wounds also likely results in fewer wound complications than saline alone.^31^

Vashe Wound Therapy contains HOCl; it was used for this study at nominal concentrations of 150 to 180 ppm. The compound is the same as the one used in the leukocyte-intracellular-killing process. Thus, Vashe Wound Therapy mimics one of the body's main ways of killing microorganisms.

We hypothesize that Vashe Wound Therapy may provide a safe, efficacious, and cost-effective alternative in burn wound management and that Vashe Wound Therapy may also reduce pain at the burn wound site.

## METHODS AND MATERIALS

This study was approved by the hospital institutional review board.

Both male and female patients, older than 18 years, and who required hospitalization were eligible for inclusion into the study. The other inclusion criterion was the presence of burn injuries that required excision and grafting, not exceeding 20% total body surface area (%TBSA).

Exclusion criteria were as follows:

- Pregnant or lactating females;
- Individuals with chlorine sensitivity; and
- Chemical, electrical, and/or frostbite injuries.

Once patients met inclusion and exclusion criteria and successfully completed the informed consent process, they were randomized to either the Vashe group or the control group. Since this was a pilot study, it aimed for 10 evaluable patients in each arm.

Outcomes evaluated in the trial were as follows:

- Graft viability (as assessed by wound inspection on every second day);
- The percentage graft take (reepithelialization) on postoperative day 14;
- Whether or not infections had occurred (as per clinical judgment and, if clinically indicated, culture results);
- Pain, assessed twice daily, both am and pm, using the Johns Hopkins visual analog assessment tool (scale 1-10); and
- The cost of the test and control materials.

Excision and grafting were performed in the standard fashion. Specifically, excision was performed tangentially with a Weck knife and hemostasis attained with epinephrine/thrombin solution and electrocautery. Split-thickness autografts were obtained with a dermatome set at 0.012-in thickness. The autografts were meshed 2:1 and secured into place with fibrin sealant and skin staples.

The grafted areas were dressed with one layer of porous silicone, which was then covered with an 8-ply burn dressing, cut to size. The 8-ply dressing was moistened with the test or control solution intraoperatively and then every 6 to 8 hours or more frequently if deemed necessary to keep the proper level of moisture. Retention dressings were cotton netting. Splints were applied as necessary.

The dressings were to be left undisturbed (except for irrigation) for a total of 5 days’ duration.

Data collected included graft take and reepithelialization, incidence of infection, pain (using the Johns Hopkins visual analog scale), cost of materials used, and adverse and serious adverse experiences.

## RESULTS

General demographic data and patient burn characteristics are shown in Table 1. A total of 19 patients participated in the trial. Eleven patients (8 males, 3 females) were in the Vashe Wound Therapy group and 8 (5 males, 3 females) were in the control group. The average age of the patients in the Vashe Wound Therapy group was 43.4 years and 53.6 years for the control group. The mean %TBSA burned was 10% in the Vashe Wound Therapy group and 6.5% in the control group. No significant differences were noted between the 2 groups.

Pain data are also shown in Table 1. Mean baseline pain level was 5.4 in the Vashe Wound Therapy group and 4.5 in the control group. These pain scores were not statistically different.

The location of the study burns is identified in Table 2. Burn location for the 2 groups was fairly equally distributed between upper and lower extremities. Two torso burns were included in the Vashe group.

Average skin graft take on postgrafting day and hospital length of stay (LOS) data are shown in Table 3. There was no difference in graft take between the Vashe and control groups (97.4% and 96.0%, respectively). The LOS was 21.6 days for the Vashe group and 15.6 days for the control group (*P* = .01).

Cost data are demonstrated in Table 4. The volume of solution used in the Vashe group was more than twice as the volume used in the control group. Even so, the hospital and patient costs were less in the Vashe group than those in the control group. When corrected for volume, Vashe therapy was much less costly than control solution.

There were 3 serious adverse events in the Vashe group and 2 in the control group. None were considered to be related to the study or to study solutions. Nonserious adverse events were also equivalent between the 2 groups. Thus, the safety profiles of the Vashe and control groups were equivalent.

## DISCUSSION

This study demonstrated that HOCl used as a topical antimicrobial solution over excised and autografted burn wounds was safe and effective compared with 5% Sulfamylon solution. Specifically, there was no difference in healing at day 14 postgrafting or in adverse events or serious adverse events.

An interesting finding was the significantly increased LOS in the HOCl group compared with the control group. The burns in the HOCl group were larger than those in the control group. No other obvious differences were noted. Also, since graft take and reepithelialization were the same in both groups, it is unlikely that the increased LOS was caused by grafting-related factors.

While the median level of pain was the same for both groups during the study, the pain at baseline was higher in the HOCl group. Thus, pain reduction in the HOCl group was better than that in the control group.

The most compelling finding in this study was the significant decrease in cost in the HOCl group compared with the control group. When size of burn and amount of solution were accounted for, the cost savings were over $406 per patient.

This was a small pilot study and was not adequately powered to provide definitive conclusions regarding safety and efficacy. A larger trial might be necessary to confirm the positive trends—equivalent efficacy and safety, better pain control, and lower costs—shown in this study.

HOCl demonstrated equivalent efficacy and safety compared with 5% Sulfamylon when used as the postoperative topical dressing for skin grafts. HOCl was more cost-effective.

## Figures and Tables

**Table 1:** Basic demographics and pain scores*

	Vashe	Control	*P*
Number	11	8	NS
Mean age, y	43.3	53.6	NS
Male, %	8	5	NS
Female, %	3	3	NS
% TBSA burned	10.0	6.5	NS
Baseline mean pain score	5.4	4.5	NS

*NS indicates nonsignificant; TBSA, total body surface area.

**Table 2:** Burn location

Vashe	Control
Right arm, shoulder, breast	Left chest, upper arm
Bilateral lower legs	Left lower leg
Bilateral lower legs	Left thigh
Abdomen	Left lateral thigh
Left leg	Right upper arm
Right flank	Right lateral thigh
Left upper extremity	

**Table 3:** Graft take at 14 days and length of hospital stay*

	Vashe	Control	*P*
Number	9	7	
Graft take, %	97.4	96.0	NS
Length of stay, d	21.6	15.6	0.01

*NS indicates nonsignificant.

**Table 4:** Volume and cost of hypochlorous acid and control solutions

	Vashe	Control
Number of patients	11	8
Per patient volume, mL	6,234.50	4,136.88
Total volume, mL	68,580	33,095
Per patient hospital cost, $	249.38	393.00
Total hospital cost, $	2,743.20	3,144.00
Total hospital cost/vol, $/mL	0.04	0.09
Per patient charge, $	1,558.60	1,965.00
Total patient charges, $	17,144.60	15,720.00
Total patient charges/vol, $/mL	0.25	0.47
